# State of the Art in Adoption of Contact Tracing Apps and Recommendations Regarding Privacy Protection and Public Health: Systematic Review

**DOI:** 10.2196/23250

**Published:** 2021-06-10

**Authors:** Katarzyna Kolasa, Francesca Mazzi, Ewa Leszczuk-Czubkowska, Zsombor Zrubka, Márta Péntek

**Affiliations:** 1 Division of Health Economics and Healthcare Management Kozminski University Warsaw Poland; 2 Queen Mary University of London London United Kingdom; 3 Maastricht University Maastricht Netherlands; 4 Kozminski University Warsaw Poland; 5 Health Economics Research Center Óbuda University Budapest Hungary; 6 Corvinus Institute for Advanced Studies Corvinus University of Budapest Budapest Hungary

**Keywords:** COVID-19, contact tracing app, data accessibility, data privacy, mobile app, digital health, digital contact tracing

## Abstract

**Background:**

During the COVID-19 pandemic, contact tracing apps have received a lot of public attention. The ongoing debate highlights the challenges of the adoption of data-driven innovation. We reflect on how to ensure an appropriate level of protection of individual data and how to maximize public health benefits that can be derived from the collected data.

**Objective:**

The aim of the study was to analyze available COVID-19 contact tracing apps and verify to what extent public health interests and data privacy standards can be fulfilled simultaneously in the process of the adoption of digital health technologies.

**Methods:**

A systematic review of PubMed and MEDLINE databases, as well as grey literature, was performed to identify available contact tracing apps. Two checklists were developed to evaluate (1) the apps’ compliance with data privacy standards and (2) their fulfillment of public health interests. Based on both checklists, a scorecard with a selected set of minimum requirements was created with the goal of estimating whether the balance between the objective of data privacy and public health interests can be achieved in order to ensure the broad adoption of digital technologies.

**Results:**

Overall, 21 contact tracing apps were reviewed. In total, 11 criteria were defined to assess the usefulness of each digital technology for public health interests. The most frequently installed features related to contact alerting and governmental accountability. The least frequently installed feature was the availability of a system of medical or organizational support. Only 1 app out of 21 (5%) provided a threshold for the population coverage needed for the digital solution to be effective. In total, 12 criteria were used to assess the compliance of contact tracing apps with data privacy regulations. Explicit user consent, voluntary use, and anonymization techniques were among the most frequently fulfilled criteria. The least often implemented criteria were provisions of information about personal data breaches and data gathered from children. The balance between standards of data protection and public health benefits was achieved best by the COVIDSafe app and worst by the Alipay Health Code app.

**Conclusions:**

Contact tracing apps with high levels of compliance with standards of data privacy tend to fulfill public health interests to a limited extent. Simultaneously, digital technologies with a lower level of data privacy protection allow for the collection of more data. Overall, this review shows that a consistent number of apps appear to comply with standards of data privacy, while their usefulness from a public health perspective can still be maximized.

## Introduction

The COVID-19 outbreak has shown how digital solutions can transform the health care system in an unprecedented manner. The rapid implementation of numerous innovative technologies to treat patients with COVID-19 has highlighted how much the human race can really benefit from data-driven transformation [1,2].

However, not all digital solutions have been launched in a contiguous manner. A specific example in this case includes mobile technologies. In principle, there are three areas in which mobile phone apps could aid in the fight against the COVID-19 pandemic: (1) self-diagnosis and facilitating treatment, (2) monitoring and enforcing the quarantine of infected persons, and (3) signaling if one is in close contact with an infected person [3].

The app used in the third area is called a *contact tracing app* and uses a smartphone to create a memory of any close contact with others for a significant amount of time. A warning is sent in case an infection is registered for anyone from the memory list, notifying the phone’s owner to get tested and possibly self-quarantine.

Despite ongoing challenges to limit the spread of the virus, the launch of such digital solutions still faces several hurdles. Meanwhile, digital contact tracing apps have enormous potential, given the fact that there are more than 3.5 billion mobile phone users worldwide. However, there is significant resistance to allowing such technologies to interfere in the lives of individuals. More than one hundred nongovernmental organizations and civil rights associations have urged governments not to use the pandemic as an excuse to enter a new era of digital surveillance [4].

In an effort to overcome potential privacy challenges in the adoption of contact tracing technologies, the European Data Protection Board (EDPD) has published guidance for the use of location data and contact tracing tools intended to mitigate the impact of the COVID-19 pandemic [3]. Moreover, Article 9 (2) of the General Data Protection Regulation (GDPR) allows for access to special categories of data if the processing of such data is necessary for reasons of public interest in the public health sector, such as protection from serious cross-border threats to health. However, such a possibility consists of neither the full compression of privacy rights nor the GDPR itself [5].

Hence, existing guidelines and principles available at the European level should be considered as safe tools to guide developers of digital solutions, even in critical times, so as to ensure the adequate respect of individuals’ rights. On the other hand, considering that the public health benefit of contact tracing apps depends directly on their widespread use, it is desirable to have governments strive to build trust among citizens with a high level of transparency. The pandemic showed how individual health is strictly connected to others’ health, and that one of the most effective measures in the absence of a vaccine is the behavior of the individual, for that one person as much as for the whole community.

Therefore, while it is desirable to develop an app that guarantees an adequate level of privacy, it is equally desirable that citizens feel the need to use such an app as an act of social responsibility toward themselves and the community in general.

In this context, the objective of our study was to address the following two questions: (1) How do available contact tracing apps allow for data collection for public health benefits and comply with standards of data privacy? and (2) Can the balance between public health interests and the protection of personal data be established to ensure the broad use of digital technologies?

The success or failure of the adoption of contact tracing apps will have impacts beyond the ongoing fight against the COVID-19 pandemic. It will set a precedent for future opportunities and challenges in the integration of other digital solutions into clinical practice while ensuring the data privacy of its users. Therefore, we hope that our conclusions and recommendations can contribute to the debate regarding the adoption of digital technologies in the support of solving health issues, even beyond challenges related to the COVID-19 pandemic. 

## Methods

### Systematic Review

We first performed a literature search in PubMed, MEDLINE, IEEE (Institute of Electrical and Electronics Engineers), and ACM (Association for Computing Machinery) Digital Library databases, covering the period between January 1 and August 31, 2020. The following key phrases were applied: “contact tracing,” “contact detector,” “contact mapping and COVID-19,” “COVID-2019,” “severe acute respiratory syndrome coronavirus 2,” “2019-nCoV,” and “SARS-CoV-2.” The search terms are included in Multimedia Appendix 1. Only research articles written in English presenting a specific contact tracing app were included. Other publications, such as news articles, editorials, commentaries, reviews, or letters, were excluded. No geographical restriction was imposed. The selection and review of included publications were conducted independently by two reviewers, and discrepancies were resolved by consensus. The PRISMA (Preferred Reporting Items for Systematic Reviews and Meta-Analyses) checklist was followed [6].

### Supplementary Search

A supplementary search on the GitHub repository, as well as on governmental and app webpages, was then performed separately to collect additional information related to contact tracing apps that were identified in the systematic literature review. The following terms were screened: (1) overview, (2) frequently asked questions (FAQ), (3) data privacy, and (4) terms of use. 

### Assessment of the Fulfillment of Public Health Interests and Compliance With Data Privacy Guidelines

In order to address the first research question, two checklists were then developed to ensure a standardized approach toward the review of each technology. Both were sourced based on external reports in the fields of interest. The first checklist was constructed to review the key functions of mobile apps that define their fulfillment of public health interests. It was based on the Ada Lovelace Institute’s report [7]. The second checklist was constructed to verify the compliance of contact tracing apps with selected data privacy standards. The following European guidelines were adopted in that respect: (1) the Privacy Code of Conduct for mobile health apps from the European Commission [8] and (2) the guidelines on the use of location data and contact tracing tools in the context of the COVID-19 outbreak from the EDPD [9]. The definition of each data privacy criterion that was used is available in Multimedia Appendix 2.

### Balance Between Data Privacy and Public Health Interests

Finally, in order to address the second research question, a set of minimum requirements that simultaneously fulfill objectives of data privacy and public health was constructed. All reviewed contact tracing apps were ranked. The score of 1 or –1 was applied for each condition being met or not met, respectively. In the absence of information, a score of 0 was assigned. All requirements were considered as equally important. The ranking of all the reviewed technologies was constructed based on the number of scores granted.

## Results

### Systematic Review and Supplementary Research Eligibility

**Figure 1:**
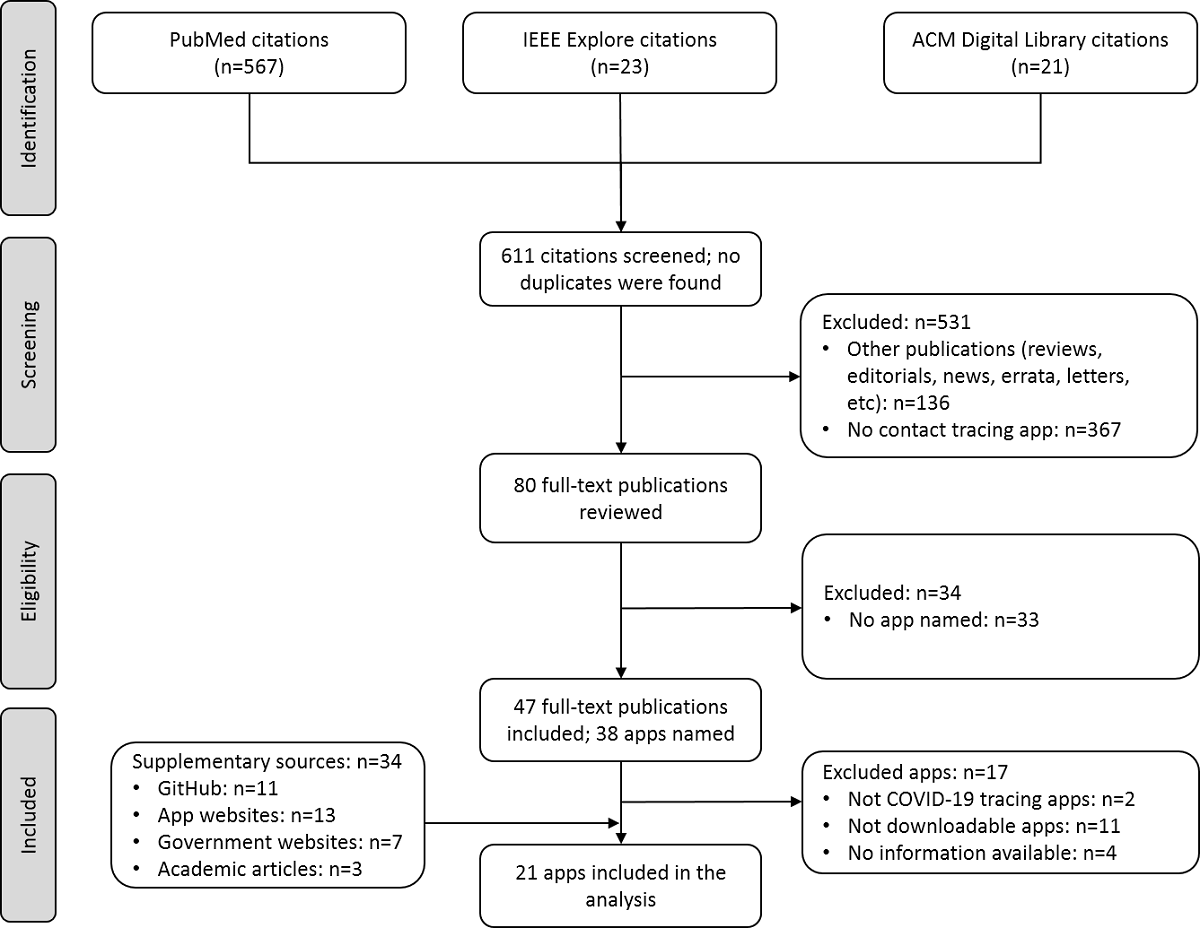
Flowchart of the article selection process for the literature search. ACM: Association for Computing Machinery; IEEE: Institute of Electrical and Electronics Engineers.

Overall, 611 unique records were identified across three databases, of which 531 articles did not meet the inclusion criteria (Figure 1). The abstract review of 80 full-text publications led to the exclusion of a further 33 articles (Multimedia Appendix 3). As the result of the full-text review of the remaining 47 publications, 38 different contact tracing apps were identified. Out of these 38 mobile apps, 17 were further excluded due to the unavailability of downloadable systems (n=11), a lack of sufficient information needed to perform the study (n=4), and not meeting criteria for contact tracing technology (n=2) (Figure 1). Further reasons for the exclusion of apps are provided in Multimedia Appendix 4 [10-42]. In the final set, 21 digital technologies were included. Among them were 18 (86%) and 3 (14%) apps that were used nationally and internationally, respectively. Additional information was collected from 34 supplementary references: government websites (n=7), app websites (n=13), academic articles (n=3), and GitHub (n=11) (Multimedia Appendix 5).

### Assessment of the Apps’ Fulfillment of Public Health Interests 

In total, 11 criteria were defined to assess how digital technologies were able to fulfill public health interests. The three most frequently adopted types of functions in the group of reviewed contact tracing apps were (1) information about geographical coverage, (2) contact alerting, and (3) governmental responsibility. The two least frequently adopted functions were (1) medical and organizational support and (2) efficiency threshold (Multimedia Appendix 6 [10-27,43-56]). Out of the 21 apps, 19 (90%) received support from governments, but only 1 (5%) provided the additional threshold required to establish an app’s efficiency. A confirmation of diagnosis with COVID-19 was required in 20 out of 21 (95%) cases, and 5 (24%) apps offered the possibility to register symptoms of COVID-19, such as fever, cough, or dyspnea. In 11 cases out of 21 (52%), the recommendation to self-isolate was issued. Other types of proximity data, such as recent travel, potentially related disorders, possible point of contact, and risk factors such as chronic disorders, were collected in half of apps as well (11/21, 52%) (Multimedia Appendix 6). Alipay Health Code was the only app that collected data about medical treatment; however, no details were found in that respect. Public health authorities and employers could access data stored in 10 (48%) and 1 (5%) contact tracing apps, respectively. Finally, there were two forms of support offered through contact tracing apps: a symptom checker and health care coordination (Multimedia Appendix 6).

### Assessment of the Apps’ Compliance With Data Privacy Guidelines

In total, 12 criteria were defined to verify to what extent digital technologies complied with data privacy guidelines. The three most frequently met conditions were (1) user consent, (2) voluntary basis, and (3) adoption of anonymization techniques (Multimedia Appendix 7 [10-27,43-56]). The provisions of information about (1) personal data breaches, (2) data gathered from children, and (3) confirmation of no data sharing to third parties were the least often implemented (Multimedia Appendix 7). As far as adequate security measures were concerned, 16 out of 21 (76%) contact tracing apps used a decentralized approach and 2 (10%) used a centralized approach. The remaining 3 (14%) apps did not provide such information. Moreover, 14 out of 21 (67%) apps published their code in the GitHub open source repository (Multimedia Appendix 7). Out of the 21 cases, 10 (48%) used anonymization techniques, with encryption methods being employed in 14 (67%) (Multimedia Appendix 7). Among other techniques, data aggregation, hashing algorithms, and asymmetrical unique keys were identified. Furthermore, 15 of the 21 (74%) apps used pseudo-random (ie, frequently changing and ephemeral) identifiers as a method to ensure cybersecurity. This method suggests that the temporary ID of each device is created and modified periodically. Bluetooth appeared to be the most common method, which was adopted in 18 of the 21 (86%) apps, while 5 (24%) used GPS only, or in addition to Bluetooth. Regarding data retention, the average period of retention of proximity data was 3 weeks (Multimedia Appendix 7). Finally, 13 out of 21 (62%) apps used a mechanism to verify COVID-19–positive results, specifically verification through a code sent to a mobile phone or confirmed by health care professionals (Multimedia Appendix 7).

### Balance Between Data Protection and Public Health Interests

The set of 10 and 6 minimum requirements related to data privacy and public health interests, respectively, were defined to assess the balance between these two domains (Multimedia Appendix 8). None of the analyzed apps managed to comply with all 16 conditions. COVIDSafe and SwissCovid met all 10 data privacy requirements and 5 out of 6 criteria for fulfillment of public health interests (15 points). The worst performance was achieved by Alipay Health Code (–3 points). Among the reviewed technologies, the standards of governmental accountability, anonymization, and encryption were the most frequently fulfilled. The establishment of an efficiency threshold and the adoption of rules against data breaches were the criteria with the lowest compliance.

## Discussion

### Principal Findings

The success of the digital revolution in the health care sector depends on access to a consistent volume of quality data that are useful to medical advancement, while ensuring an appropriate level of protection of personal data privacy. In our research, we attempted to systematically analyze the state of the art in the adoption of contact tracing apps in the fight against the COVID-19 pandemic, with the objective to verify to what extent the requirements concerning data protection and public health interests can be achieved simultaneously.

In order to develop the comprehensive list of criteria for our analysis, we searched the public domain for available guidelines in the fields of interest. We adopted a human-centered approach in that respect, which should be interpreted from two perspectives.

Firstly, when addressing the type of data being collected, we strived to choose the most comprehensive set of criteria that would ensure the maximization of public health benefit from a societal perspective. Therefore, we deliberately selected the Ada Lovelace Institute’s report, which is based on the core values and the underlying mission of the institute (ie, to ensure that data and artificial intelligence work for people and society) [7].

Secondly, when addressing data protection, we focused on guidelines that were developed based on the most comprehensive set of data protection rules, such as the GDPR. From the European perspective, data protection is, in fact, considered a fundamental human right. Hence, we selected (1) the Privacy Code of Conduct on mobile health apps from the European Commission [8] and (2) the guidelines on the use of location data and contact tracing tools in the context of the COVID-19 outbreak from the EDPD. The GDPR is a regulation (ie, directly effective for member states without the need of further national laws) that concerns all types of processing of personal data and it applies to entities that process the personal data of European citizens. Hence, compliance with the GDPR is to be considered implicit for all European apps. The two selected legal instruments have a different level of authority, being a set of guidelines and a code of conduct, which means that developers can voluntarily commit to follow the rules. Hence, we decided to evaluate to what extent such rules—that we considered relevant in relation to contact tracing apps—have been followed by developers. Let us note that contact tracing apps can be considered mobile health technologies for two reasons: (1) information about COVID-19 positivity equates to health data and (2) there often are other consequences when the app sends a notification about contact with a positive case (eg, the receiver has a recommendation to self-isolate, whereas others need to contact health care providers). Hence, these consequences may be equivalent to the notion of *providing health advice*.

Not only did we develop the set of criteria for our analysis based on the chosen guidelines, but we also constructed a scorecard that simultaneously evaluated compliance with data protection and the level of fulfillment of public health benefits. It should be noted that although we did assign the same weight to all minimum requirements on the scorecard, we do value data protection as an essential condition due to the recognition of privacy as a human right and the urge to prevent a surveillance society. Hence, from our perspective, meeting the data protection criteria should be recommended as the mandatory base upon which to build digital health solutions and, consequently, public health benefits.

In sum, we reviewed 37 records about 21 contact tracing apps. In total, 12 and 11 criteria were used for the assessment of compliance with data privacy and fulfillment with public health interests, respectively. There are three key important findings worth highlighting.

Firstly, the majority of reviewed contact tracing apps were, to a greater extent, compliant with data privacy standards. As many as 18 out of 21 (86%) apps were designed to be used on a voluntary basis. Such an approach should be considered as optimal because it prevents any discrimination against individuals who are unable or unwilling to download the app. Moreover, the mandatory use of digital technology could lead to financial and technical burdens. Hence, jurisdictions with mandatory use of such apps are actually not compliant with the European standards. As demonstrated in Multimedia Appendix 7, BeAware Bahrain, Alipay Health Code, and Aarogya Setu were part of this category. A consistent number of apps used the decentralized approach (16/21, 76%), which is preferable to the centralized approach from a cybersecurity perspective. Additionally, Bluetooth technology with changing ephemeral identifiers was employed in 15 out of 21 (71%) cases, which is preferable to GPS for privacy reasons, as Bluetooth is less intrusive. Still, there is room for improvement with respect to compliance with data privacy; in addition, the rules against data breaches were provided in only 3 out of 21 (14%) cases.

Secondly, as far as public health interests are concerned, there is still room for improvement among contact tracing apps. Only 8 out of 21 (38%) apps provided the definition of close contact, and governmental accountability was limited to 19 out of 21 (90%) cases. Additionally, 10 out of 21 (48%) apps reviewed solutions that were allowed for data accessibility for health care professionals. In that group, only 8 out of 10 (80%) apps provided details regarding techniques used for data aggregation, encryption, and/or anonymization. Meanwhile, there were just 13 out of 21 (62%) apps that used the mechanism of diagnosis verification, and the threshold for the population coverage needed for the digital solution to be effective was provided only once. 

Thirdly, we observed the tendency that, with a high level of compliance with data privacy regulations, public health interests could be achieved to a limited extent, whereas a lower level of compliance with data privacy occurred across cases with greater potential for data collection. The COVIDSafe and SwissCovid apps met 15 out of 16 requirements. Both ensured that the minimum amount of data necessary for contact tracing was collected (ie, proximity data through Bluetooth). Contrary to that, Alipay Health Code ranked last in the scorecard as it processed a vast amount of data. Such a difference seems to be linked with different cultural, geographic, and political backgrounds. In principle, collecting more data could be useful from a public health perspective, for example, in order to closely monitor the contagion or to have a clearer and bigger picture of the spread of the disease from an epidemiological standpoint. Nonetheless, in relation to Alipay Health Code, no information was found about the actual use of the collected data for public health purposes. The lack of information available to users concerning the use of their data for further public health purposes would not, per se, infringe on the European principles as expressed in the GDPR. Indeed, Article 5.1 (b) of the GDPR considers further processing of collected data for public interest as compatible with the initial purpose of processing. Nonetheless, from a policy perspective, such ambiguity about the use of personal data might not be desirable in the European environment. In fact, not only might it disincentivize the use of apps due to the lack of transparency, but it could also have detrimental effects on society by undermining trust between citizens and governments.

Our findings need to be regarded with caution due to certain limitations of our study that must be acknowledged. Firstly, given the dynamic pandemic situation, we are aware that new developments in the field occur on a daily basis; as such, a number of new contact tracing apps were published after our systematic literature review was completed, or they were excluded due to limited information. On that note, it is worth mentioning that one of the most downloaded apps in the United States, namely HealthLynked COVID-19 Tracker [57,58], was not included in our study due to a lack of accessible information. Indeed, no sufficient information in relation to privacy and public health conditions was available in order to include the app in our tables. In fact, the website of the developer does not provide technical details nor a dedicated privacy policy for the tracking app; only the general privacy policy for other pre-existing HealthLynked apps was found. In our view, such disconnection between information provided to users and users’ downloads of the app raises serious privacy concerns and does not constitute a good practice for future developers.

Secondly, let us highlight that our checklists were based on specific references, while there have been other relevant guidelines already developed in relation to COVID-19 contact tracing apps. An example in this case is the article *Ethical guidelines for COVID-19 tracing apps*, which was already published in *Nature* by a group of researchers from the University of Oxford after our study had been completed [10]. Such guidelines include not only principles of privacy but also relevant ethical issues, such as equality in the access to digital technology, which we do not discuss in our publication. 

Thirdly, we deliberately omitted the assessment of effectiveness of reviewed contact tracing apps, as this was already published by other authors [59].

Finally, although we used a global approach to search for apps, our analysis was conducted from a European perspective. Non-European jurisdictions have diverse cultural and political backgrounds and, as such, may follow different sets of values [60]. Even before the COVID-19 outbreak, it had been routine to undergo temperature checks before visiting public places, maintain social distancing, and wear masks in some non-European countries [60]. Their technological apparatus is entirely different from the European one, as is the level of citizens’ trust for the government. Accordingly, solutions adopted in such countries are not necessarily easily applicable in European settings and vice versa [60].

### Conclusions

Despite the above limitations, we hope that our results and conclusions will inspire approaches on how to develop digital health solutions and ensure their broad adoption. Our literature review indicated that a consistent number of apps appear to be substantially compliant with the standards of data privacy, while the usefulness of contact tracing technologies from the public health perspective can still be maximized. Available surveys indicate that attitudes toward the use of contact tracing apps vary across different jurisdictions. According to a University of Oxford survey, the acceptance rate for contact tracing apps ranged from 67.5% to 85.5% in France, Germany, and Italy [61]. Conversely, a Pew Research Center study found that 60% of Americans believe that location tracking will not help limit the spread of COVID-19, and only 45% believe that such an app is allowed to track citizens who have had contact with an infected person [62]. Still, available estimates indicate that more than half of the population should use such an app in order to provide public health benefits [63]. Therefore, the question is how to ensure the broad adoption of contact tracing apps when their adoption happens on a voluntary basis, as was the case in 18 out of the 21 (86%) reviewed technologies. At the same time, only 1 of the 21 (5%) reviewed contact tracing apps provided information regarding the threshold for its efficiency in combatting the COVID-19 pandemic. Consequently, the ultimate objective can be defined as the need to establish an evidence-based approach to the definition of the *broad use* of contact tracing apps and an educational campaign about their benefits. Indeed, mandatory use of digital health solutions would undermine the right to personal freedom that characterizes democratic societies. Another recent global survey on 7804 respondents from seven countries revealed that as many as 41% of the study population indicated data security as the number one barrier to adopting digital solutions. The adoption of the decentralized approach, which was the case for 16 out of 21 (76%) apps, may provide a better chance to obtain the required trust among end users [64].

Overall, a prima facie analysis suggests that the more intrusive the government is into an individual’s privacy, the more that public health can benefit from the data. Moreover, it seems that a high level of privacy protection corresponds to an obstacle in terms of the use of data for public health. Nonetheless, we argue that the two interests can be perceived as not contradictory. Indeed, digital health solutions that protect individuals’ privacy can be directed toward optimization of public health benefits. To achieve such a goal, the adoption of transparency policies that increase trust between public and private stakeholders should be encouraged. In fact, solid public confidence in digital solutions developed by governments that prioritize the protection of the rights of individuals can foster further data sharing for public health purposes, among other things.

Indeed, we believe that the key success factors in this matter are transparency and information campaigns targeting individuals, which can be achieved by working toward an awareness of citizens’ responsibility and by providing relevant information on data protection and cybersecurity. Fostering citizen involvement in public matters such as public health can help to make every individual feel like a member of the state. Such awareness would stimulate individuals to share their data in a controlled and safe environment for the benefit of society.

## Appendix

Multimedia Appendix 1 Search terms for the systematic review.

Multimedia Appendix 2 Definitions of data privacy standard criteria.

Multimedia Appendix 3 Reasons for exclusion of full-text articles.

Multimedia Appendix 4 Reasons for exclusion of apps.

Multimedia Appendix 5 Supplementary sources of information.

Multimedia Appendix 6 Assessment of public health interests.

Multimedia Appendix 7 Assessment of data privacy.

Multimedia Appendix 8 Ranking of apps.

## Abbreviations

ACM: Association for Computing Machinery
EDPD: European Data Protection Board
EIPIN IS: European Intellectual Property Institutes Network–Innovation Society
FAQ: frequently asked questions
GDPR: General Data Protection Regulation
IEEE: Institute of Electrical and Electronics Engineers
PRISMA: Preferred Reporting Items for Systematic Reviews and Meta-Analyses
