# Supplementary material for: State of the Art in Adoption of Contact Tracing Apps and Recommendations Regarding Privacy Protection and Public Health: Systematic Review
Source: JMIR Mhealth Uhealth. 2021 Jun 10;9(6):e23250. doi: 10.2196/23250 (PMC8195202; doi:10.2196/23250)
Supplement: Multimedia Appendix 1 [file mhealth_v9i6e23250_app1.docx]

**Multimedia Appendix 1: Search Terms**

| **Database** | **Search Term** |
| --- | --- |
| Pubmed | ("contact tracing"[All Fields] OR "contact detector"[All Fields] OR "contact mapping"[All Fields]) AND ("COVID-19"[All Fields] OR "covid 2019"[All Fields] OR "severe acute respiratory syndrome coronavirus 2"[All Fields] OR "2019-nCoV"[All Fields] OR "SARS-CoV-2"[All Fields])  Filters: from 01/01/2020- 31/08/2020 |
| IEEE Explore | ((((((("All Metadata":"contact tracing") OR "All Metadata":"contact detector") OR "All Metadata":"contact mapping and COVID-19") OR "All Metadata":"COVID-2019") OR "All Metadata":"severe acute respiratory syndrome coronavirus 2") OR "All Metadata":"2019-nCoV") OR "All Metadata":"SARS-CoV-2")  Filter: year:2020; Journals;  Publication dates selected: 01/01/2020- 31/08/2020 |
| ACM Digital Library | [All: "contact tracing"] OR [All: "contact detector"] OR [All: "contact mapping and covid-19"] OR [All: "covid-2019"] OR [All: "severe acute respiratory syndrome coronavirus 2"] OR [All: "2019-ncov"] OR [All: "sars-cov-2"] AND [Publication Date: (01/01/2020 TO 31/08/2020)]  Filter: Research Article |
