# Supplementary material for: State of the Art in Adoption of Contact Tracing Apps and Recommendations Regarding Privacy Protection and Public Health: Systematic Review
Source: JMIR Mhealth Uhealth. 2021 Jun 10;9(6):e23250. doi: 10.2196/23250 (PMC8195202; doi:10.2196/23250)
Supplement: Multimedia Appendix 2 [file mhealth_v9i6e23250_app2.docx]

| **Multimedia Appendix 2. Definitions** | |  |
| --- | --- | --- |
| **Table** | **Criterion** | **Definition** |
| 2 | Governmental responsibility | Government is one of parties that is responsible for the application and recommendation of its use. |
| 2 | Definition of close contact | Definition of what a close contact means in case of using the application. Guidelines from the federal Centers for Disease Control and Prevention define ‘close contact’ as anyone who has been within around 2 meters of an infected person's secretions. |
| 2 | Efficiency threshold | Coverage of population which installed the application to maximise effectiveness. |
| 2 | Geographical coverage | Location where the application is in use. |
| 2 | Data collected | Types of information collected by an application. |
| 2 | Data sharing | Sharing information collected by an application. |
| 2 | Infection reporting | User and/or institution report infection in an application after a COVID-19 positive result. |
| 2 | Contact alerting | Users and/or institutions are informed about the infection. |
| 2 | Actions if positive result of COVID-19 | Action taken in case of infection. |
| 2 | Organisational support provided | Organisational assistance provided by the state in case of infection. |
| 2 | Medical support provided | Medical assistance provided by the state in case of infection. |
| 3 | Centralised vs Decentralised | Centralised approach - data with information about possible infection are stored on the company's servers. Decentralised - data are stored on the user device, and after the infection is diagnosed, information is sent to the server. |
| 3 | Contact tracing using Bluetooth / GPS | Bluetooth as Low Energy methods without user location data. Through GPS application collect information about user's location. |
| 3 | Anonymisation techniques | Anonymisation - general information; encryption - process of encoding information; hashing algorithm/data aggregation/asymmetrical unique keys - detailed methods of encryption. |
| 3 | Pseudo-random identifiers | It is a process of creating and refreshing encrypted temporary user ID. |
| 3 | Voluntary basis | The government does not require to download and install the application. |
| 3 | Data retention policy | Information on how long data are stored. |
| 3 | Verification of COVID-19 positive result | Authorisation and confirmation of COVID-19 positive result by healthcare authorities using special single-code. |
| 3 | User consent | The user must consent to the information about the infection and the transfer of data to third parties. |
| 3 | Information about personal data breach | Application provides the details of what personal data breach is, how to protect against it or what actions are taken subsequently. |
| 3 | Data gathered from children (under 18) | Data gathering needs to be considered in relation to the age of the child and their skills and capabilities. |
| 3 | Sharing data to third parties | Information if application sends data to other subjects (third parties). |
| 3 | Access to source code (GitHub repository) | Everyone can see the application's development code and download it. |
