# Supplementary material for: State of the Art in Adoption of Contact Tracing Apps and Recommendations Regarding Privacy Protection and Public Health: Systematic Review
Source: JMIR Mhealth Uhealth. 2021 Jun 10;9(6):e23250. doi: 10.2196/23250 (PMC8195202; doi:10.2196/23250)
Supplement: Multimedia Appendix 3 [file mhealth_v9i6e23250_app3.docx]

**Multimedia Appendix 3: Reasons for exclusion of full-text articles**

| **Source** | **Author, year** | **Reason for exclusion** | **Reference** |
| --- | --- | --- | --- |
| PubMed | Korea Centers for Disease Control and Prevention, 2020 | No application named | doi: 10.24171/j.phrp.2020.11.1.09 |
| PubMed | Häfner SJ, 2020 | No application named | doi: 10.1016/j.bj.2020.08.008 |
| PubMed | Chen CM, 2020 | No application named | doi: 10.2196/19540 |
| PubMed | Jung F, 2020 | No application named | doi: 10.3233/CH-209004 |
| PubMed | Mahmood S, 2020 | No application named | doi: 10.2196/18980 |
| PubMed | Ram N, 2020 | No application named | doi: 10.1093/jlb/lsaa023 |
| PubMed | Kretzschmar ME, 2020 | No application named | doi: 10.1016/S2468-2667(20)30157-2 |
| PubMed | Jahnel T, 2020 | Not English | doi: 10.1055/a-1195-2474 |
| PubMed | Parker MJ, 2020 | No application named | doi: 10.1136/medethics-2020-106314 |
| PubMed | Wang S, 2020 | Concept | doi: 10.2196/19457 |
| PubMed | Cheng W, 2020 | Concept | doi: 10.2196/20369 |
| PubMed | He Z, 2020 | No application named | doi: 10.1186/s12916-020-01551-8 |
| PubMed | Iyengar KP, 2020 | No application named | doi: 10.1016/j.ijtb.2020.07.014 |
| PubMed | Altmann S, 2020 | No application named | doi: 10.2196/19857 |
| PubMed | Cioffi A, 2020 | No application named | doi: 10.1016/j.jemep.2020.100575 |
| PubMed | Lee D, 2020 | No application named | doi: 10.1016/j.worlddev.2020.105057 |
| PubMed | Bengio Y, 2020 | No application named | doi: 10.1093/jamia/ocaa153 |
| PubMed | Arakpogun EO, 2020 | No application named | doi: 10.1016/j.worlddev.2020.105105 |
| PubMed | Bianconi A, 2020 | No application named | doi: 10.1088/1478-3975/abac51 |
| PubMed | Collado-Borrell R, 2020 | No application named | doi: 10.2196/20334 |
| PubMed | Smith CD, 2020 | No application named | doi: 10.5888/pcd17.200246 |
| PubMed | Klenk M, 2020 | No application named | doi: 10.1007/s10676-020-09544-0 |
| PubMed | Nijsingh N, 2020 | No application named | doi: 10.1007/s11673-020-10004-z |
| PubMed | Zhang Y, 2020 | No application named | doi: 10.1016/j.ajic.2020.03.012 |
| PubMed | Lenert L, 2020 | No application named | doi: 10.1093/jamia/ocaa039 |
| PubMed | Kapa S, 2020 | No application named | doi: 10.1016/j.mayocp.2020.04.031 |
| PubMed | Ivers LC, 2020 | No application named | doi: 10.1016/S2468-2667(20)30160-2 |
| PubMed | Bengio Y, 2020 | No application named | doi: 10.1016/S2589-7500(20)30133-3 |
| PubMed | Park S, 2020 | No application named | doi: 10.1001/jama.2020.6602 |
| PubMed | Yamamoto K, 2020 | Concept | doi: 10.2196/19902 |
| PubMed | Ghinita G, 2020 | Concept | doi: 10.1007/s10707-020-00410-1 |
| PubMed | Alqutob R, 2020 | No application named | doi: 10.2196/19332 |
| IEEE | Adans-Dester CP, 2020 | No application named | doi: 10.1109/OJEMB.2020.3015141 |
| ACM | Fitzsimons JK, 2020 | No application named | doi: 10.1145/3407023.3409204 |
