# Supplementary material for: State of the Art in Adoption of Contact Tracing Apps and Recommendations Regarding Privacy Protection and Public Health: Systematic Review
Source: JMIR Mhealth Uhealth. 2021 Jun 10;9(6):e23250. doi: 10.2196/23250 (PMC8195202; doi:10.2196/23250)
Supplement: Multimedia Appendix 4 [file mhealth_v9i6e23250_app4.docx]

**Multimedia Appendix 4. Reasons for exclusion of applications**

| **Application name, reference** | **Reason for exclusion** |
| --- | --- |
| CarePredict PinPoint [50] | No information available |
| CommCare [22] | Not app (system) |
| ConTra Corona [21] | Not app (protocol) |
| CoronApp [19,29] | No information available in English |
| Covid Symptom Tracker [41] | Not contact tracing app (symptom study) |
| DP3T [21,37,42,51-54] | Not app (protocol) |
| EpiOne [21] | Not app (protocol) |
| Google-Apple [12,19-22,26,29,30,37,39-44,46,48,52-62] | Not app (protocol) |
| HealthLynked [37] | No technical information available |
| PACT (East Coast) [21] | Not app (protocol) |
| PACT (West Coast) [21] | Not app (protocol) |
| PEPP-PT [22,29,40,42,44,54,63] | Not app (protocol) |
| Pronto-C2 [21] | Not app (protocol) |
| Real-time location system vs EMR review in hospital [64] | Not app (system) |
| ROBERT [46] | Not app (protocol) |
| StayHomeSafe [19] | Not contact tracing app (quarantine) |
| Tracking App [33] | No information available |
