# Supplementary material for: State of the Art in Adoption of Contact Tracing Apps and Recommendations Regarding Privacy Protection and Public Health: Systematic Review
Source: JMIR Mhealth Uhealth. 2021 Jun 10;9(6):e23250. doi: 10.2196/23250 (PMC8195202; doi:10.2196/23250)
Supplement: Multimedia Appendix 5 [file mhealth_v9i6e23250_app5.docx]

**Multimedia Appendix 5. Supplementary sources of information**

| **Application** | **Supplementary source of information (URL / DOI)** | **Source** |
| --- | --- | --- |
| Aarogya Setu | https://www.mygov.in/aarogya-setu-app/ | Government website |
| ABTrace Together | https://www.alberta.ca/privacystatement.aspx | Government website |
| Alipay/WeChat | https://doi.org/10.1007/s11845-020-02215-5  <https://doi.org/10.1186/s12942-020-00202-8>  https://doi.org/[10.2196/19359](https://doi.org/10.2196/19359) | Academic article  Academic article  Academic article |
| BeAware | https://apps.bahrain.bh/CMSWebApplication/action/ShowAppDetailsAction?selectedApp ID =321&appLanguage=en | Application website |
| COCOA | https://www.mhlw.go.jp/stf/seisakunitsuite/english_pp_00032.html | Application website |
| Corona-Warn-App | https://www.coronawarn.app/en/ https://github.com/corona-warn-app | Application website Github |
| COVID Trace | https://nvhealthresponse.nv.gov/covidtrace/ | Government website |
| COVIDSafe | https://www.covidsafe.gov.au/ https://github.com/AU-COVIDSafe/mobile-android | Government website Github |
| CovidWatch | https://www.covid-watch.org/ https://github.com/covidwatchorg/covidwatch-android-tcn | Application website Github |
| GH Covid-19 Tracker | https://ghcovid19.com | Application website |
| HaMagen | https://govextra.gov.il/ministry-of-health/hamagen-app/download-en/ https://github.com/MohGovIL/hamagen-react-native | Government website  Github |
| Immuni | https://www.immuni.italia.it/ https://github.com/immuni-app | Application website Github |
| NHS Covid-19 App | https://apps.apple.com/gb/app/nhs-covid-19/id1520427663 | Application website |
| Private Kit: Safe Paths (other name: COVID Safe Paths) | https://safepaths.mit.edu/ https://github.com/Path-Check/safeplaces-dct-app | Application website Github |
| ProteGO | https://www.gov.pl/web/cyfryzacja/protego-safe--pobierz-zainstaluj-przetestuj https://github.com/ProteGO-Safe/android | Application website  Github |
| Smittestopp | https://www.helsenorge.no/en/smittestopp/ https://github.com/folkehelseinstituttet/Fhi.Smittestopp.App | Application website Github |
| StopCovid (new version TousAnitCovid) | https://bonjour.tousanticovid.gouv.fr/index-en.html | Government website |
| Stopp Corona | https://www.roteskreuz.at/site/meet-the-stopp-corona-app/  https://github.com/austrianredcross/stopp-corona-android | Application website Github |
| Swiss Covid | https://www.bag.admin.ch/bag/en/home/krankheiten/ausbrueche-epidemien-pandemien/aktuelle-ausbrueche-epidemien/novel-cov/swisscovid-app-und-contact-tracing.html https://github.com/DP-3T/dp3t-app-android-ch | Application website Github |
| Tabaud | https://tabaud.sdaia.gov.sa/IndexEn | Application website |
| Trace Together | https://www.tracetogether.gov.sg/ https://github.com/opentrace-community/opentrace-android | Government website Github |
