# Supplementary material for: State of the Art in Adoption of Contact Tracing Apps and Recommendations Regarding Privacy Protection and Public Health: Systematic Review
Source: JMIR Mhealth Uhealth. 2021 Jun 10;9(6):e23250. doi: 10.2196/23250 (PMC8195202; doi:10.2196/23250)
Supplement: Multimedia Appendix 6 [file mhealth_v9i6e23250_app6.docx]

**Multimedia Appendix 6. Assessment of the Fulfillment of Public Health Interests**

| **#** | **Name** | **Governmental responsibility** | **Definition of close contact** | **Efficiency threshold** | **Geographical coverage** | **Data collected** | **Data sharing** | **Infection reporting** | **Contact alerting** | **Actions if COVID-19 positive result** | **Organizational support provided** | **Medical support provided** |
| --- | --- | --- | --- | --- | --- | --- | --- | --- | --- | --- | --- | --- |
| 1 | Aarogya Setu [19-23] | Yes | Less than 2m | ·· | India | COVID-19 positive/ negative; general health; personal data; travel history; time; citizen location information | ·· | Self-reported | To user | Quarantine for employees | ·· | ·· |
| 2 | ABTrace Together [24] | Yes | 2 meters | ·· | Alberta | COVID-19 positive/ negative; phone number; information | User and public health authorities | Self-reported | To user and medical professionals | Report exposures to the Ministry of Health | Contact from contact tracers | guidance and information to minimize further transmission. |
| 3 | Alipay/WeChat [12,22,25-32] | Yes | ·· | ·· | People's Republic of China | Symptoms of COVID-19; medical treatment; isolated observation; possible contacts; health status; travel history; passport details; QR-codes | User and public health authorities | Self-reported and by medical professionals | To user and medical professionals | Quarantine | ·· | ·· |
| 4 | BeAware [19] | Yes | ·· | ·· | Bahrain | COVID-19 positive/ negative; possible contacts; citizen location information; mobile number | User only | Self-reported | To user | Self-quarantine | ·· | Health recommendations |
| 5 | COCOA [33] | Yes | 1 meter – 15 minutes or more | ·· | Japan | COVID-19 positive/ negative | User and public health authorities | Self-reported | To user and medical professionals | Report exposures to the Ministry of Health |  | symptom checker provided in the app to receive appropriate guidance such as contact information of healthcare organizations. Depending on the symptoms, you will be advised to take a COVID-19 test. You will also find instructions if you want to reach out healthcare organizations by phone. |
| 6 | Corona-Warn-App [28,33,34] | Yes | ·· | ·· | Germany | COVID-19 positive/ negative; symptoms of COVID-19; QR-codes | User and public health authorities | Self-reported | To user and/or medical professionals | Self-quarantine | ·· | ·· |
| 7 | COVID Trace [29] | Yes | .. | ·· | Nevada | COVID-19 positive/ negative; | Users only | Self-reported | To users | Basic directions provided (isolate, test) | .. | .. |
| 8 | COVIDSafe [12,19,21,28,35-37] | Yes | 1.5m and minimum 15 minutes | ·· | Australia | COVID-19 positive/ negative; encrypted reference code; date, time and proximity of contacts; registration data (name, mobile phone number, age range and post code) | User and public health authorities | ·· | To user and medical professionals | Decided by medical professionals | ·· | ·· |
| 9 | CovidWatch [21,37] | No | ·· | 56% of population overall or 80% of all smartphone users using the app | Worldwide | COVID-19 positive/ negative; date | User only | Self-reported | To user | ·· | ·· | ·· |
| 10 | GH Covid-19 Tracker [19,28] | Yes | ·· | ·· | Ghana | COVID-19 positive/ negative; citizen location information; health related information; general information (sex, age, name, phone number, risk factors, region) | User and public health authorities | Self-reported | To user and medical professionals | Self-quarantine | ·· | ·· |
| 11 | HaMagen [19,21] | Yes | Minimum 15 minutes | ·· | Israel | COVID-19 positive/ negative; citizen location information | User only | Self-reported and by medical professionals | To user and medical professionals | Report exposures to the Ministry of Health/self-quarantine | ·· | ·· |
| 12 | Immuni [29] | Yes | Less than 2m and minimum 15 minutes | ·· | Italy | COVID-19 positive/ negative | User and public health authorities | Self-reported | To user | Self-quarantine | ·· | ·· |
| 13 | NHS Covid-19 App [12,29,38,39] | Yes | .. | ·· | England, Wales, Scotland, Northern Ireland, Jersey and Gibraltar | COVID-19 positive/ negative; postcode; symptoms; QR codes of visited venues; | Users and public health authorities | Self-reported and automatically by medical professionals if test booked with the app | To users | Recommendation to self-isolate | Alert: Lets you know the level of coronavirus risk in your postcode district.  Check-in: Use our simple QR code scanner to check-in to venues like bars and restaurants. You will get alerted if you have visited a venue where you may have come into contact with coronavirus.  Symptoms: Check if you have coronavirus symptoms and see if you need to order a test.  Test: Helps you order a test if you need to.  Isolate: Keep track of your self-isolation countdown and access relevant advice. | .. |
| 14 | Private Kit: Safe Paths [21,23,40-45] | No | ·· | ·· | Worldwide | COVID-19 positive/ negative; symptoms of COVID-19; | User only | Self-reported | To user | Self-quarantine | ·· | Health recommendations |
| 15 | ProteGO [22,26] | Yes | ·· | ·· | Poland | COVID-19 positive/ negative; health status | User and public authorities | Self-reported | To user | Decided by medical professionals | ·· | ·· |
| 16 | Smittestopp [12] | Yes | Contact with someone infected lasted more than about 15 minutes | ·· | Norway | COVID-19 positive/ negative; symptoms of COVID-19; | User only | Self-reported | To user | Recommendations on what to do next | ·· | Health recommendations |
| 17 | StopCovid (new version TousAnitCovid) [21,26,33,46] | Yes | ·· | ·· | Worldwide | COVID-19 positive/ negative; proximity history; Country codes; data entered into the application (when tested positive) | User and public health authorities | Self-reported | To user | ·· | ·· | ·· |
| 18 | Stopp Corona [19,26,28,47] | Yes | Less than 2m and minimum 15 minutes | ·· | Austria | COVID-19 positive/ negative; approximate citizen location information | User only | Self-reported and by medical professionals | To user and medical professionals | Decided by medical professionals and/or self-quarantine | ·· | ·· |
| 19 | Swiss Covid [21,28,33] | Yes | Less than 1.5m and minimum 15 minutes | ·· | Switzerland | COVID-19 positive/ negative | User only | Self-reported | To user | Self-quarantine | ·· | Health recommendations via call center |
| 20 | Tabaud [22] | Yes | ·· | ·· | Saudi Arabia the United Kingdom, USA | COVID-19 positive/ negative | User and public health authorities | Self-reported and by medical professionals | To user and/or medical professionals | Report exposures to the Ministry of Health/self-quarantine | ·· | Health recommendations |
| 21 | Trace Together [12,19,21-23,27,29,30,35,37,44,48,49] | Yes | ·· | ·· | Singapore | COVID-19 positive/ suspect; phone number; device information | User only | Self-reported and by medical professionals | To user and medical professionals | Voluntary report exposures to the Ministry of Health | Contact from Ministry of Health | ·· |

·· – no information in searched resources
