# Supplementary material for: State of the Art in Adoption of Contact Tracing Apps and Recommendations Regarding Privacy Protection and Public Health: Systematic Review
Source: JMIR Mhealth Uhealth. 2021 Jun 10;9(6):e23250. doi: 10.2196/23250 (PMC8195202; doi:10.2196/23250)
Supplement: Multimedia Appendix 7 [file mhealth_v9i6e23250_app7.docx]

**Multimedia Appendix 7. Assessment of the Compliance with Data Privacy Guidelines**

|  | **Name** | **Centralised vs Decentralised** | **Contact tracing using Bluetooth / GPS** | **Anonymisation techniques** | **Pseudo-random identifiers** | **Voluntary basis** | **Data retention policy** | **Verification of COVID-19 positive result** | **User consent** | **Information about personal data breach** | **Data gathered from children (under 18)** | **Sharing data to third parties** | **Access to source code (GitHub repository)** |
| --- | --- | --- | --- | --- | --- | --- | --- | --- | --- | --- | --- | --- | --- |
| 1 | Aarogya Setu [19-23] | Centralised | Bluetooth, GPS | Encryption, anonymisation | ·· | No (for all employees and citizens living in containment zones) | 30 days, 45 days (no tested positive), 60 days (tested positive) | ·· | Yes | ·· | ·· | ·· | No |
| 2 | ABTrace Together [24] | Decentralised | Bluetooth | Encryption | Yes | Yes | 21 days | Yes | Yes | ... | ·· | ·· | Yes |
| 3 | Alipay/WeChat [12,22,25-32] | ·· | ·· | ·· | ·· | No | ·· | ·· | Maybe | ·· | ·· | ·· | No |
| 4 | BeAware [19] | ·· | GPS | Encryption | ·· | No (for quarantined cases) | 14-42 days | ·· | Maybe | ·· | ·· | Yes | No |
| 5 | COCOA [33] | Decentralised | Bluetooth | Encryption | Yes | Yes | 14 days | Yes | Yes | ... | ·· |  | Yes |
| 6 | Corona-Warn-App [28,33,34] | Decentralised | Bluetooth | Encryption, anonymisation, hashing algorithm | Yes, each 10-20 minutes | Yes | 14 days | Yes | Yes | ·· | Yes, above age 16 | No | Yes |
| 7 | COVID Trace [29] | Decentralised | Bluetooth | Encryption | Yes | Yes | 14 days | Yes | Yes | ... | ·· | No | No |
| 8 | COVIDSafe [12,19,21,28,35-37] | Decentralised | Bluetooth | Encryption | Yes, each two hours | Yes | 21 days | Yes | Yes | Yes | Yes, above age 16 with authorization of one parent | ·· | Yes |
| 9 | CovidWatch [21,37] | Decentralised | Bluetooth | Anonymisation | Yes, short intervals | Yes | 14-28 days | Yes | Yes | ·· | Yes, above age 13 with authorisation of one parent | Yes | Yes |
| 10 | GH Covid-19 Tracker [19,28] | ·· | Bluetooth, GPS | ·· | ·· | Yes | ·· | ·· | Yes | ·· | ·· | No | No |
| 11 | HaMagen [19,21] | Decentralised | Bluetooth, GPS | Encryption | ·· | Yes | 30 days | Yes | Yes | Yes | ·· | ·· | Yes |
| 12 | Immuni [29] | Decentralised | Bluetooth | Encryption, anonymisation, data aggregation | Yes, several times each hour | Yes | no later than December 2020* | Yes | Yes | ·· | Yes, above age 14 with authorisation of one parent | No | Yes |
| 13 | NHS Covid-19 App [12,29,38,39] | Decentralised | Bluetooth | Encryption, anonymisation | Yes | Yes | 28 days (contact codes only) | Yes | Yes | ·· | ·· | No | Yes |
| 14 | Private Kit: Safe Paths [21,23,40-45] | Decentralised | GPS | Encryption | ·· | Yes | 14 days | ·· | Yes | ·· | ·· | ·· | Yes |
| 15 | ProteGO [22,26] | Decentralised | Bluetooth | Anonymisation | Yes, each 10 minutes | Yes | 14 days | ·· | Yes | ·· | No | No | Yes |
| 16 | Smittestopp [12] | Decentralised | Bluetooth | Anonymisation | Yes, each 10-20 minutes | Yes | 14 days | Yes | Yes | ·· | Yes, above age 16 | No | Yes |
| 17 | StopCovid (new version TousAnitCovid) [21,26,33,46] | Centralised | Bluetooth | Anonymisation | Yes | Yes | 14 days; 180 days (tested positive) | Yes | Yes | ·· | ·· | ·· | No |
| 18 | Stopp Corona [19,26,28,47] | Decentralised | Bluetooth | Encryption, asymmetrical unique keys | Yes | Yes | 7 days (digital handshake contacts), 14 days (metadata), 30 days (tested positive) | ·· | Yes | ·· | ·· | ·· | Yes |
| 19 | Swiss Covid [21,28,33] | Decentralised | Bluetooth | Encryption, anonymisation | Yes | Yes | 14 days | Yes | Yes | Yes | Yes, above age 12 | ·· | Yes |
| 20 | Tabaud [22] | Decentralised | Bluetooth | Anonymisation | Yes | Yes | 14 days | ·· | Yes | ·· | Yes | ·· | No |
| 21 | Trace Together [12,19,21-23,27,29,30,35,37,44,48,49] | Decentralised | Bluetooth | Encryption, anonymisation | Yes | Yes | 21 days | Yes | Yes | ·· | ·· | No | Yes |

·· – no information in searched resources
